# Richardson's Mechanical Dentistry

**Published:** 1897-05

**Authors:** 


RICHARDSON'S MECHANICAL DENTISTRY. Seventh Edition. Six hundred and ninety Illustrations. Revised, enlarged and edited by George W. Warren, D. D. S., Chief of Clinical Staff, Pennsylvania College of Dental Surgery. Publishers, P. Blakiston, Son, & Co., Philadelphia.

The object of the editor in preparing the seventh edition of this well known and highly appreciated text book on prosthetic dentistry, has been to make it pre-eminently practical for students and young practitioners, and also that it may answer as an exponent of the present status of dental prosthesis. That he has succeeded, a perusal of the new edition clearly shows, as much of the text has been rewritten, three new chapters added, and new systems and appliances introduced, and without materially increasing the dimensions of the book so as to render it inconvenient. Many of the illustrations are new and from original drawings, and useless methods and obsolete theories have been eliminated.

The three new chapters added to this seventh edition, comprise "Application for the Correction of Fractured Maxillæ (Interdental Splints); "Appliances for the Correction of

Dental Irregularities"; and "Electricity and its Application in Dental Meehanics," all of which are useful, instructive and interesting.

The publishers are to be commended for the excellence of style, arrangement and printing of this work, all of which are superior to those of the former editions, and make a volume attractive and convenient, and well adapted for a standard text book.

